# Assessing data availability and quality within an electronic health record system through external validation against an external clinical data source

**DOI:** 10.1186/s12911-019-0864-2

**Published:** 2019-07-25

**Authors:** Ellen L. Palmer, John Higgins, Saeed Hassanpour, James Sargent, Christina M. Robinson, Jennifer A. Doherty, Tracy Onega

**Affiliations:** 1 Dartmouth College, Hanover, NH USA; 2 Dartmouth College, Hanover, NH USA; 3 Dartmouth College, Hanover, NH USA; 4 Dartmouth College, Hanover, NH USA; 5 New Hampshire Colonoscopy Registery, Lebanon, NH USA; 6 Huntsman Cancer Institute, Salt Lake City, UT USA; 7 Dartmouth College, Hanover, NH USA

**Keywords:** Smokers registry, Informatics pipeline, Electronic health records, Natural language processing

## Abstract

**Background:**

Approximately 20% of deaths in the US each year are attributable to smoking, yet current practices in the recording of this health risk in electronic health records (EHRs) have not led to discernable changes in health outcomes. Several groups have developed algorithms for extracting smoking behaviors from clinical notes, but none of these approaches were assessed with external data to report on anticipated clinical performance.

**Methods:**

Previously, we developed an informatics pipeline that extracts smoking status, pack year history, and cessation date from clinical notes. Here we report on the clinical implementation performance of our pipeline using 1,504 clinical notes matched to an external questionnaire.

**Results:**

We found that 73% of available notes contained no smoking behavior information. The weighted Cohen’s kappa between the external questionnaire and EHR smoking status was 0.62 (95% CI 0.56–0.69) for the clinical notes we were able to extract information from. The correlation between pack years reported by our pipeline and the external questionnaire was 0.39 on the 81 notes for which this information was present in both. We also assessed for lung cancer screening eligibility using notes from individuals identified as never smokers or smokers with pack year history extracted by our pipeline (*n* = 196). We found a positive predictive value of 85.4%, a negative predictive value of 83.8%, sensitivity of 63.1%, and specificity of 94.7%.

**Conclusions:**

We have demonstrated that our pipeline can extract smoking behaviors from unannotated EHR notes when the information is present. This information is reliable enough to identify patients most likely to be eligible for smoking related services. Ensuring capture of smoking information during clinical encounters should continue to be a high priority.

## Background

Smoking is the leading cause of preventable illness and death in the US and around the globe, costing $170 billion dollars a year in healthcare spending [1] and $150 billion in productivity loss due to smoking related illness [2] in the US alone. From a population standpoint, approximately 20% of all US deaths [3–5] and 28% of cancer-specific deaths are attributable to smoking [6], explaining in part these high costs. In an attempt to reduce the burden of smoking on health outcomes, smoking status and other smoking behaviors [7] are frequently collected during clinical appointments.

However, this information is not utilized in a systematic fashion to improve patient outcomes. Instead, the burden of identifying smoking behaviors and referring patients to smoking cessation counseling, educational services, preventive care, and appropriate disease screening is left to clinicians with limited time. This leads to short or non-existent critical conversations about tobacco addiction and associated health risks so that the primary visit reason can be properly addressed. Automated identification of smoking behaviors within the electronic health records (EHR) could improve identification of patients eligible for smoking related services, generate lists for specialized teams, and reduce the overall burden on clinicians.

To create such a system, health informatics researchers have been working on developing smokers’ registries to collect smoking information from multiple EHR sources into a database format [8–10]. The two most common information sources are semi-structured fields and clinical notes. Semi-structured fields, such as the drop-down boxes for smoking status, were previously used for accountable care reporting and are both common and an easy data source to access. Clinical notes can be used to generate a probability-based smoking status, typically using natural language processing (NLP) algorithms [11, 12]. These algorithms perform well within the system in which they are trained, but usually require retraining or modification when tested in an external system [13]. One weakness of most of these methods is that they focus exclusively on identifying smoking status. Additional information relating to pack year history and, where applicable, most recent cessation date is needed to determine lung cancer screening eligibility [14], provide appropriate pre-surgical counseling [15, 16] and determine readiness to quit [17]. Without these pieces of information, a smokers’ registry will be severely limited in terms of clinical usefulness.

Other deficits in knowledge related to a smoker’s registry development include a lack of reported concordance between semi-structured and NLP identified smoking behaviors and validation of EHR findings against an external source. Current validations of NLP methods are limited to reporting how well the algorithm performs against human-annotated labels instead of an external ground truth. Here, we address these specific knowledge-gaps by assessing a previously reported NLP-pipeline for concordance with semi-structured EHR data and against an external health questionnaire.

## Methods

### Questionnaire and EHR data

The New Hampshire Colonoscopy Registry (NHCR) is a state-wide colonoscopy registry which has been collecting comprehensive colonoscopy data from patients and endoscopists across New Hampshire and conducting research on many aspects of colorectal cancer screening through colonoscopy, since 2004 [18]. Adult patients presenting for a colonoscopy for any reason at participating sites across New Hampshire were invited to enroll in the registry. Consenting patients completed a questionnaire which included questions about smoking status, number of packs per day, and number of years smoked [18]. All patients were between 50 and 80 years old at the time of their NHCR registration and consented to participate in research studies.

We received smoking-related responses and demographic information on a sample of 3,000 NHCR participants who completed registry enrollment at a Dartmouth-Hitchcock site. We extracted the clinical note and semi-structured smoking behaviors data from the visit date closest to the completion of the NHCR questionnaire for each patient, provided it was not the same visit at which the questionnaire was completed. Participant were excluded from further analyses if they did not have another visit date.

All participants were 18 years or older and provided written informed consent. Both this study and the NHCR were approved by the Dartmouth College Committee for the Protection of Human Subjects.

### Natural language processing pipeline development

We used clinical notes extracted from the local Epic EHR data warehouse in April 2016 to develop and evaluate our informatics pipeline. This set included 533 annotated notes to train the algorithms, and 223 annotated notes to validate the algorithms. The development and reporting of standard metrics for our pipeline are presented in a companion paper [19] and repository [20].

In summary, annotations of the 756 clinical notes used to develop the algorithm was completed by the first author. The smoking status algorithm we used adapts an algorithm first presented by Cohen et al. [12]. In summary, this method is a multi-class support vector machine, with classifications of “current”, “former”, “smoker temporality unknown”, “never”, and “unknown”. Classification vectors were selected based on the presence of a ‘hotspot’ word. Our adaptations included reducing the hotspot set from six to four roots (“smok”, “cig”, “tobac”, and “nicoti”) and including +/− 5 words around the hotspot instead of a set 100 characters. Multiple hotspots identified in a single note were appended together to classify each note at the document level.

To identify pack year history, we applied a regular expression algorithm to sentences which included the stems “cig”, “smok”, “pk”, “ppd”, “pack”, “pak”, or “pkyr”. These sentences were parsed to identify if one or two numbers (as numerical or word entries) were present. If two numbers were present, the reporting was classified as containing both frequency and duration. Where the frequency was reported as cigarettes per day, we converted it to packs per day by assuming 20 cigarettes per pack. The two numbers were then multiplied together to report a pack year history. If a single number was present, further processing was completed to determine if the number was frequency, duration, or actual pack years smoked.

Finally, to identify cessation dates, sentences containing “quit”, “qd”, or “stop” were extracted. If a date was found in the hotspot-identified sentence, it was converted to a uniform format using the Python package “datetime”.

### Statistical assessments and reporting

The NLP pipeline, using 533 annotated records to train the support vector machine classifier [19], was applied to the NHCR-matched clinical notes to identify smoking status, pack year histories, and cessation dates.

In cases where NLP pipeline-identified and semi-structured data were both available, we assessed the concordance of smoking status labels and correlation of pack year histories between the two EHR sources.

**Fig. 1:**
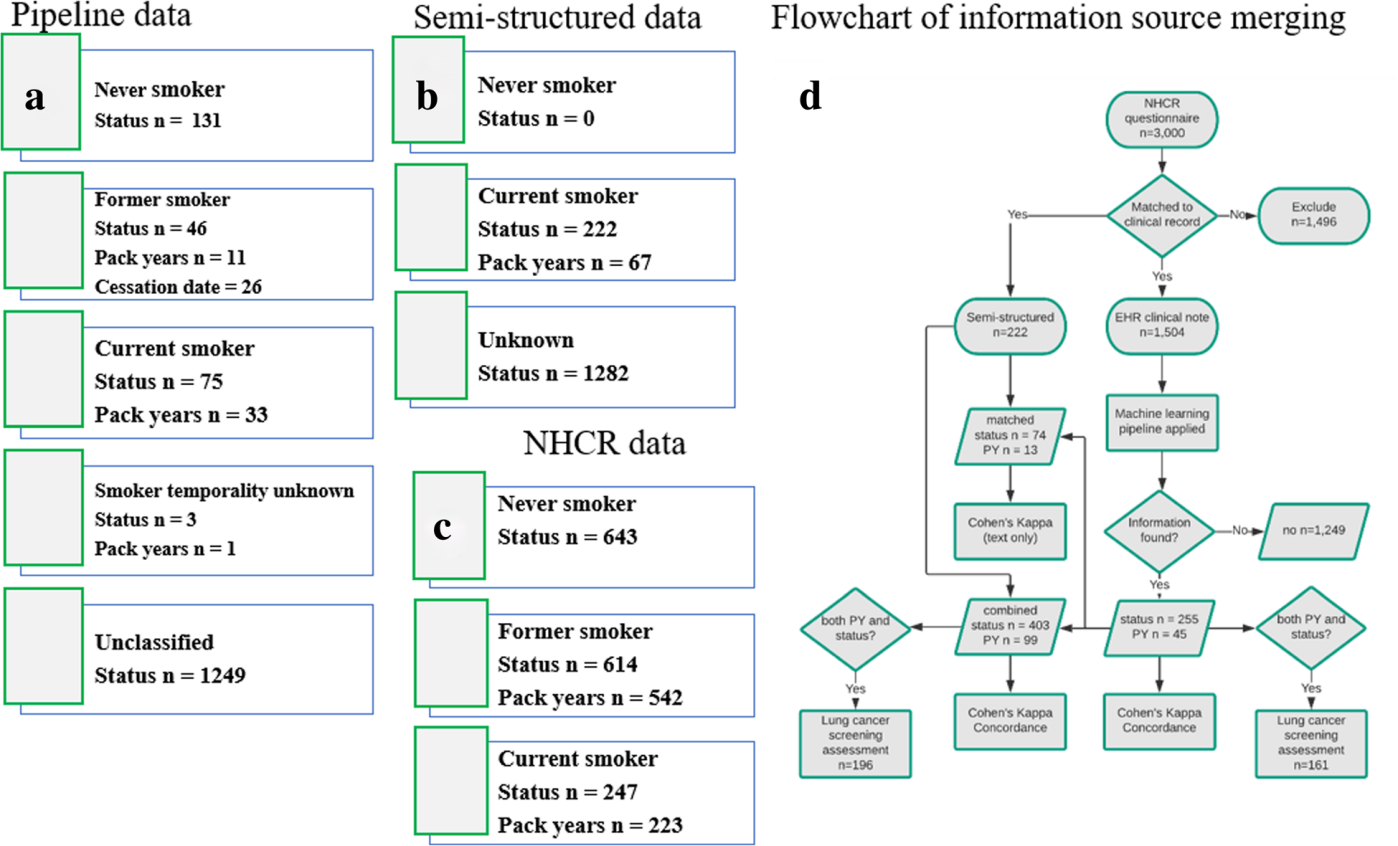
Data Availability and Linkage Process. Information sources utilized to assess the quality of the informatics pipeline for smoking behavior data. **a** Breakdown of information captured by the informatics pipeline **b** Breakdown of information from the semi-structured EHR fields **c** Breakdown of information from the NHCR and **d** Workflow of the information sources utilized, and how they relate to each other. Abbreviations: Electronic Health Record (EHR), New Hampshire Colonoscopy Registry (NHCR), Pack years (PY)

To assess the NLP pipeline against an external source, Cohen’s weighted kappa and concordance statistics were calculated comparing the NLP pipeline identified smoking status to the NHCR reported status. To determine if there was an improvement when we included the semi-structured data, we also calculated Cohen’s weighted kappa and concordance for the NHCR status against the combined pipeline and semi-structured statuses. In cases of discordant status labels between the two EHR sources, we kept the worst-label, ordered by current > former > smoker temporality unknown > never > unknown. For instance, if a patient was labeled a current smoker in the semi-structured data and a smoker temporality unknown by the NLP-pipeline, we retained the current smoker label. Similarly, we retained the highest pack year estimate from the two sources when reporting the correlation between pack year histories in the joint-EHR data and NHCR questionnaire. A flowchart summarizing this process for merging the NLP pipeline and the semi-structured information in to a final dataset can be found in Fig. 1d.

### Usage case: lung Cancer screening identification

Finally, we assessed the readiness of our pipeline for a complex real-world application by identifying patients eligible for lung cancer screening based on the criteria of current or former smoker with a greater than a 30-pack year history. We report the positive predictive value (PPV), negative predictive value (NPV), sensitivity, and specificity of our pipeline in identifying screening-eligible patients, under the assumption that the NHCR responses reflects true eligibility. Due to the lack of cessation date information in the NHCR, we were unable to restrict to former smokers who quit within the prior 15 years, as recommended by current screening guidelines. Therefore, we assumed all former smokers with a 30 or more pack year history were eligible [14].

The informatics pipeline was coded using Python version 2.7 [21]. All statistics were calculated in R studio using R version 3.1.1 [22].

## Results

### Data availability

A total of 1,504 out of 3,000 NHCR participants were successfully matched to a clinical note. Of these, 75 were classified as “current”, 46 as “former”, three as “smoker temporality unknown”, 131 as “never”, and 1,249 as “unknown” by our NLP pipeline. We also identified pack year histories in 45 clinical notes (Fig. 1a). Semi-structured EHR smoking status, recorded on the same date as the clinical note, was available for 222 of the 1,504 participants. Pack-year history was available for 67 participants (Fig. 1b). From the NHCR questionnaire, the distribution of smoking statuses for the 1,504 matched individuals was 247 current, 614 former, and 643 never smokers. Of the smokers, 72 former and 24 current smokers declined answering questions regarding packs per day or years smoked questions on the questionnaire. Thus, we had pack year histories for a total of 765 out of 861 smokers (Fig. 1c).

### Concordance between semi-structured and NLP-identified smoking behaviors

Overall, 74 records had smoking status in both the NLP pipeline and semi-structured fields. All the semi-structured statuses were for current smokers, limiting our assessment of concordance between EHR sources. Fifty-two out of 74 records (71.2%) were concordant for current smoker status. The discordant records had NLP pipeline classifications of former smoker (*n* = 6), smoker temporality unknown (*n* = 1), and never smoker (*n* = 15).

Only 13 records had pack year history recorded in both sources, making a statistical analysis unreliable. Our qualitative check indicates that the pipeline underestimated pack year history nearly half the time (6 out of 13 had NLP pipeline pack year histories of five or less despite the semi-structured reporting > 25). For the other seven records, the Pearson correlation coefficient was 0.70.

### Questionnaire vs. EHR sources

The Cohen’s weighted kappa between the external questionnaire smoking status and our pipeline-identified status was 0.56 (95% CI 0.46–0.65) for 252 notes and the concordance was 63.9%. Between the questionnaire and our combined EHR data sources (pipeline and semi-structured) for 400 notes, the weighted kappa was 0.62 (95% CI 0.56–0.69) and the concordance was 67.8%. Finally, when we collapsed our smoking categories to ever/never, the Cohen’s kappa was 0.59 (95% CI 0.51–0.68) and concordance was 83.6% for 403 records. The summary statistics of these assessments can be found in Table 1, and the cross-tabulation of the label classes from the joint EHR-data sources can be found in Table 2.

**Table 1:** Smoking status and lung cancer screening eligibility identification in the EHR using an informatics pipeline

Concordance between NHCR reported status and smokers’ registry status			
	Pipeline: Clinical notes only (*n* = 252)	Pipeline and Semi-structured (*n* = 400)	Ever smoker vs. Never smoker (*n* = 403)
Cohen’s Kappa	0.56	0.62	0.59
Concordance	63.9%	67.8%	83.6%

Summary statistics for smoking status detection using the informatics pipeline on clinical notes only, the pipeline merged with semi-structured data, and the merged pipeline with semi-structure data simplified to ever smoker vs. never smoker

**Table 2:** Smoking status classification between the external questionnaire and the joint EHR sources

		EHR smoking status					Total
		Current	Former	Never	Smoker	Unknown	
External smoking status	Current	**160**	9	9	1	68	247
	Former	55	**31**	27	1	500	614
	Never	23	6	**80**	1	533	643
	Total	238	46	116	3	1,101	1,504

Cross-tabulation of the smoking statuses individuals reported on an unrelated questionnaire against our informatics pipeline identified smoking statuses. Current = current smoker; former = former smoker; never = never smoker; smoker = smoker temporality unknown; unknown = record did not contain enough information relating to smoking behaviors to classify. Bolded numbers are concordant for status between the EHR and external records

**Fig. 2:**
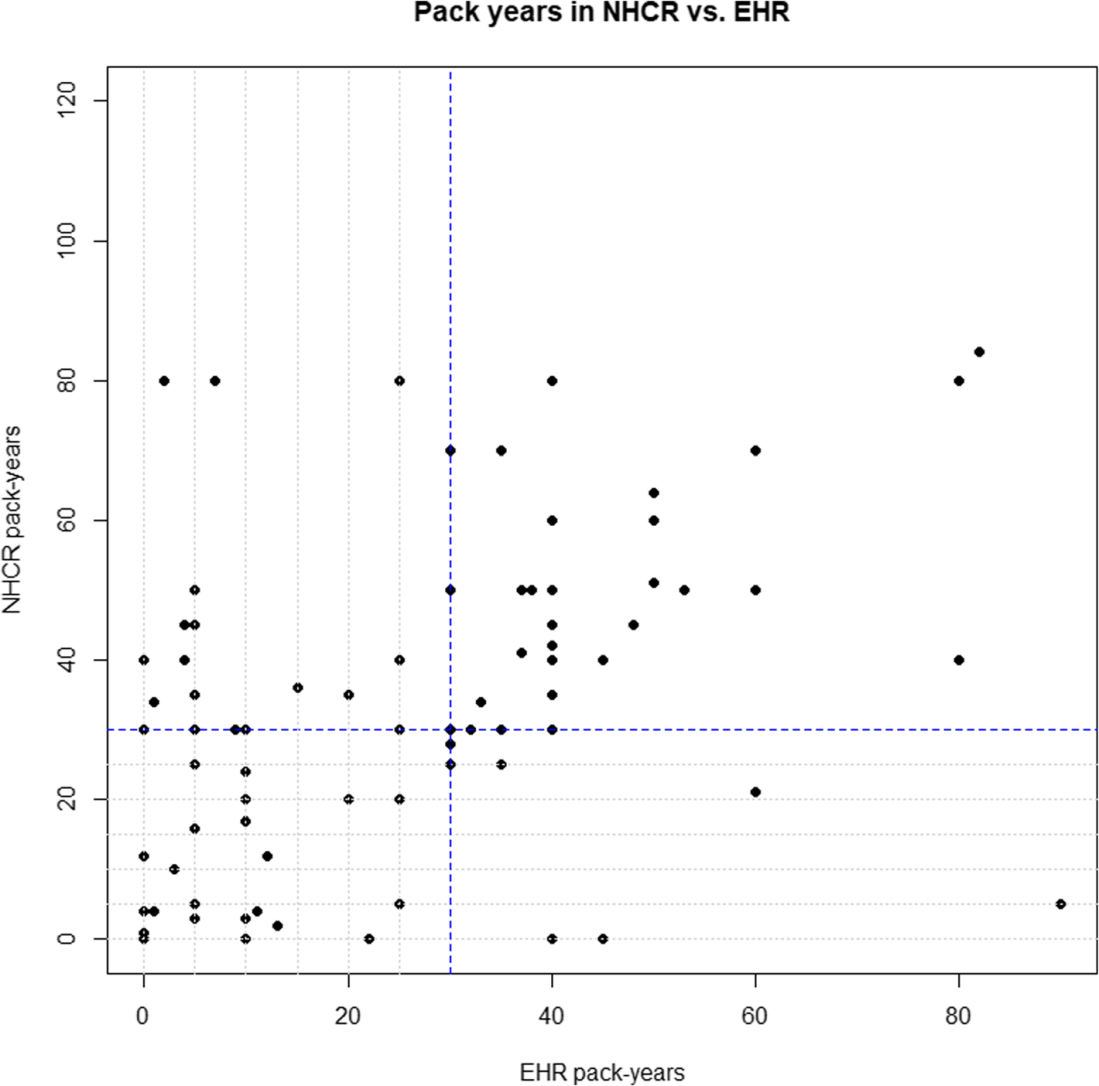
Plot of Pack Year History Reporting to External Questionnaire vs. EHR. Pack year history reported to NHCR (y-axis) against EHR derived pack year history (x-axis). Blue dashed lines indicate a 30-pack year history, the threshold for lung cancer screening. Gray dotted lines are at every 5 years, up to the 30-year line

The correlation between the NLP pipeline and the external questionnaire reported pack years was − 0.12 for the 36 records with both NLP and questionnaire reported histories (data not shown). Between the semi-structured and the external questionnaire, the correlation between reported pack years was 0.60 for 56 records (data not shown). Finally, the correlation between the combined EHR sources and the external questionnaire reported pack years was 0.39 (*n* = 81). Figure 2 shows the correlation plot of the combined EHR sources vs. the external questionnaire.

### Usage case: lung cancer screening identification

Due to the external questionnaire not reporting cessation dates, we were unable to test the cessation date algorithm against an external source; therefore, we assumed all former smokers had quit within the past 15 years for our use case. A manual review of the records with a pipeline-identified date confirmed that the extracted dates matched the clinical note dates in 21 of 26 cases.

From the NHCR questionnaire, we found 329 current and former smokers with a 30+ pack year history, making them eligible for lung cancer screening. An additional 1,079 participants were not eligible. We could not assess 96 participants due to no pack year information.

From the combined EHR information sources, we identified 54 people who were eligible for lung cancer screening based on the EHR information and 161 individuals were not eligible. We could not assess 1,283 records (missing status *n* = 1,101, status present but missing pack year history *n* = 188). The cross-tabulation of these individuals can be found in Table 3.

**Table 3:** Lung cancer screening eligibility classification between the external questionnaire and EHR sources

		EHR lung cancer screening eligibility			Total
		Eligible	Not eligible	Missing data	
External lung cancer screening eligibility	Eligible	41	24	264	329
	Not eligible	7	124	948	1,079
	Missing data	6	13	77	96
	Total	54	161	1289	1504

Lung cancer screening eligibility prediction based on external reporting and EHR derived smoking status and pack year history

Restricting to those individuals whom we could classify in both the NHCR questionnaire and EHR, we found a PPV of 85.4% (41/48), NPV of 83.8% (124/148), sensitivity of 63.1% (41/65), and specificity of 94.7% (124/131) (Table 4). When we restricted to the NLP identified information only, we note a significant loss in sensitivity (Table 4).

**Table 4:** Lung cancer screening eligibility

	Pipeline: Clinical notes only (*n* = 161)	Pipeline and Semi-structured (*n* = 196)
PPV	75.0%	85.4%
NPV	77.2%	83.8%
Sensitivity	20.9%	63.1%
Specificity	97.5%	94.7%

Summary statistics for identifying patients eligible for lung cancer screening using EHR data. These metrics assumed true eligibility was captured by the NHCR questionnaire

## Discussion

We assessed the potential application of a smokers’ registry informatics pipeline [19] integrating semi-structured and NLP-identified smoking behaviors against external data to assess the potential real world application. Our results indicate modest agreement (kappa = 0.62) between the EHR and an external questionnaire for assessment of smoking status. Due to heterogenous reporting, capturing pack year histories from clinical notes was found to be less reliable than semi-structured data. However, even with the application of our pipeline, only about one-third of the EHR records assessed contained information relation to smoking status, and even fewer contained information relating to other smoking behaviors.

One of the most striking findings of this study was the lack of data within the EHR. We requested data on 3,000 NHCR participants and planned to match text notes to +/− 6 months of the NHCR questionnaire date. Unfortunately, few records met this criterion and we dropped the temporal alignment requirement. Even so, we were unable to match nearly half of our participants to EHR records. We later identified this as a problem resulting from a significant number of participants enrolling in the NHCR before our hospital system adopted Epic in 2011 or patients being seen by our clinicians for the colonoscopy only.

We also restricted our study to semi-structured data and notes from the same date to minimize temporal errors in the comparison between the pipeline and semi-structured data. This was due to the expectation that more semi-structured would be available due to the meaningful use criteria for Medicare and Medicaid reimbursement. However, this field is only required to be updated once every 2 years.

Despite having smaller sample sizes than initially planned, this is the first study designed to assess an informatics pipeline against external data. We demonstrated that such tools, while still in need of further refinement, could have meaningful real-world applications to improve health services delivery to high risk patients. This is best demonstrated by our assessment of identifying individuals eligible for lung cancer screening, a service currently used by only about 3% of eligible patients [23]. While the sensitivity of our pipeline alone was low, incorporating semi-structured data improved identification to a level that is likely clinically actionable, capturing 85% of screening-eligible patients for whom we had enough data to assess (Table 3, Table 4).

While not our primary aim, we demonstrate that an informatics pipeline applied to unstructured text may, with the implementation of some heuristic rules, fill in data missing from semi-structured fields. We used our pipeline to identify smoking status on 252 individuals, 178 for whom semi-structured data was lacking. This information was modestly concordant with the external information, even in the absence of semi-structured data (Cohen’s kappa = 0.56 for status, Table 1). Further we found that among individuals listed as current smokers in semi-structured fields, 71.2% were also listed as current by our pipeline. These findings are important, as the support the integration of these two data sources, a more cost-effective approach than applying the pipeline to all records.

We also provided pack year estimates for 38 patients for whom this information was not available in semi-structured fields. However, here we note significant limitations. Some of the extracted pack year histories were extremely low due to misclassification of partial history information as full pack year information. We suggest a heuristic cutoff between 5 and 15 pack year, filtering out values lower than this number as these often reflect only frequency (packs per day) or duration (years smoked). Further refinement of the pack year algorithm to properly filter out partial smoking history without a heuristic cutoff is needed to improve sensitivity below this threshold. Still, we are the first to report on an algorithm that can extract pack year information from unannotated free-text clinical notes. Our code is available with this paper for others to use and improve upon.

As has been noted in other institutions, the absence of important information within the EHR can be highly prevalent [24] and remains a challenge; the exact missingness rate of smoking behaviors beyond status has, to our knowledge, not been assessed. Additional work by others is also needed to determine if there are alternative to those we propose for improving smoking behavior identification in existing records.

Despite the challenge of missing eligible individuals due to missingness or the slight tendency of our algorithm to classify down (current smokers to former, former to never), this study suggests that when information related to smoking behaviors is present we can leverage that data to improve clinical workflow. By better identifying subpopulations of patients who benefit from targeted information and intervention strategies, we can improve the delivery of pertinent health information while managing the cost and personnel time needed to implement effective screening, disease prevention, and smoking cessation services [15, 25–30]. Current practice encourages clinicians to use the ask-advise-connect model [31], determine lung cancer screening eligibility, and go through shared decision making within a visit [32]. However, physicians may not be fully trained on how to complete these processes [33–35], or find that those eligible for services have more pressing concerns that need to be addressed in the limited time clinicians have with patients [36]. Individuals automatically identified as potentially eligible for smoking-related services could be sent shared decision-making paperwork before a clinical visit or referred to tobacco experts for immediate follow up, empowering them to be proactive in preventative and early detection care.

Further work on the integration of these algorithms within EHRs may lead to customizable tools in the future, such as creating daily lists for cessation experts alerting them about patients attending the clinic for routine appointments who may benefit from a warm handoff [37, 38]. Administrators could benefit from these data as well by being able to proactively identify areas of need within their hospital catchment area based on the known health risks associated with smoking, informing future hospital resource allocation.

Even individuals with missing information could benefit from these proposed improvements of hospital systems. Individuals whose records lack partial or complete smoking behavior histories could be sent a prompt to fill in demographics information on the EHR web-portal before their next visit that includes questions about smoking. In collecting smoking behaviors information from the patients before visits, additional questions could be included to assess quit-readiness, allowing tobacco cessation coaches to be more fully prepared to connect with patients immediately after a clinical appointment. Shifting the burden of cessation counseling to specialists using these methods would allow patients and clinicians to more fully address a patient’s primary reason for seeking medical treatment while also working towards reducing unhealthy behaviors [25, 29, 30, 37, 39–44].

Given these potential future benefits, there are limitations to the work presented here. This analysis is only a demonstration of the feasibility of a potential product. Future work assessing computational needs, cost, and fully developing usage cases for an integrated system need to be completed. This study is also limited by our cross-sectional design; future work using longitudinal data remains to be completed. We also note that our pipeline often underestimates pack years due to mistaking frequency or duration information as full pack year history. This forces us to recognize the need to use heuristics to reduce errors in implementation until more sophisticated algorithms are developed and validated. Further, while our cohort is larger than any previously used for assessing lung cancer screening [45], the attrition of numbers we faced was unexpected and thus the sample size is smaller than originally planned. And while we found that our smoking status algorithm performed similarly in the Integrating Biology and the Bedside (i2b2) test set when trained on our local data (data not shown), we have not validated the entire pipeline on an external hospital system. Finally, there is a risk that by utilizing a cancer screening questionnaire for our external information, the patients included in this study are not fully representative of the patients encountered in a standard clinical setting, which could limit generalizability.

Still, utilizing an external questionnaire tests the reliability of a smoking informatics pipeline applied to an EHR against a presumed ground truth rather than just against the knowledge we know is there from abstraction. Further, since the NHCR questionnaire was completed for something unrelated to smoking, we believe patients were unlikely to be as self-conscious about their smoking behaviors as they would be in a survey designed to capture this information [46]. Another strength is that while our pipeline has some difficulty with classifying former smokers, this problem is easily correctable. First, patients and clinicians often use the term ‘former smoker’ differently, as many quit attempts fail [47–49]. Since those identified as current smokers would be included in the registry, and those incorrectly classified as never smokers in this cross-sectional study are likely to be accurately reclassified when we move to whole-record assessment, few people are likely to be detrimentally impacted in the long run. Further, by merging the pipeline information with semi-structured data, we can ‘fact-check’ the algorithm’s findings as the registry is built. Another benefit of our pipeline’s tendency to be more conservative is that never smokers are rarely classified as smokers, and those incorrect classification was usually due to generic instructions (ex: “if you currently smoke”). Minor modifications to our existing pipeline could filtering out these phrases and improve future classification.

Future directions include external validation and whole-record classification of records to reduce the number of patients with missing data, assess consistency in reporting over time, and identify other use cases for our smokers’ registry. Further, we proposed to develop the software needed to fully integrate this pipeline in to both ours and external EHRs. We plan to release our trained pipeline and interface through Observational Health Data Sciences and Informatics (OHDSI) [50] once we have finished this work to efficiently distribute our pipeline for free to other institutes. We will also continue to collaborate with clinicians and researchers to develop a comprehensive list of potential uses, identify what reports should be generated and for whom, and how those reports can aid clinicians in being more proactive with smokers to prevent or reduce the impact of smoking on health.

## Conclusions

We have built an informatics pipeline for creating a smokers’ registry from patient data within the EHR system. Further, we have validated the information quality against an external questionnaire and demonstrated a usage case. While the PPV (85.4%), NPV (83.1%), and sensitivity (61.2%) of this usage case were modest, the specificity (94.8%) ensures that resources are directed at the correct audience. Improving data capture by either clinicians or by creating a patient data entry portal could reduce the level of missingness noted in this study, while also improving the performance of tools such as automated identification of patients potentially eligible for lung cancer screening. From this work, we have found that an EHR-based smokers’ registry using clinically collected data is feasible and could improve routine care by improving patient identification for cessation and screening services without adding additional time burdens to the clinical team.

## Data Availability

The EHR dataset curated from Dartmouth-Hitchcock records is not deidentified and cannot be released due to HIPAA regulations. However, this data is available for on-site review and usage after IRB approval (contact corresponding author for additional details / arrangement of approval). The NHCR dataset was created from a collaboration with the New Hampshire Colonoscopy Registry is not deidentified and cannot be released. Summary statistics for smoking histories for the 1,504 records matched can be made available upon request to the corresponding author. Usage of the original data files upon IRB approval may be possible (contact corresponding author for additional details / arrangement of approval). All are code is freely available through a Bitbucket repository (https://bitbucket.org/ellelnutter/tobbacco_user_registery_network_public/).

## Abbreviations

EHR: Electronic Health Record
i2b2: integrating Biology and the Bedside
NLP: Natural language processing
NPV: Negative Predictive Value
OHDSI: Observational Health Data Sciences and Informatics
PPV: Positive Predictive Value
PY: Pack year
